# Anti-inflammatory effects of Neurotoxin-Nna, a peptide separated from the venom of Naja naja atra

**DOI:** 10.1186/1472-6882-13-86

**Published:** 2013-04-15

**Authors:** Yeping Ruan, Li Yao, Bingbing Zhang, Shuijuan Zhang, Jianyou Guo

**Affiliations:** 1 College of Pharmaceutical Sciences, Zhejiang Chinese Medical University, Hangzhou, 310053, P.R China; 2 Key Laboratory of Mental Health, Institute of Psychology, Chinese Academy of Sciences, Beijing, 100101, P.R China

**Keywords:** Neurotoxin-Nna, Anti-inflammatory, Antinociceptive, Tumor necrosis factor alpha, Interleukin 1 beta, Nuclear factor kappa B

## Abstract

**Background:**

Neurotoxin-Nna (NT), an analgesic peptide separated from the venom of Naja naja atra, has reported to have an exceptional specificity to block transmission of the nerve impulse by binding to the α- subunit of the nicotinic acetylcholine receptor in the membrane. However, little information is available on the anti-inflammatory effects of NT. Therefore, the anti-inflammatory activity of Neurotoxin-Nna was investigated in this study.

**Methods:**

The anti-inflammatory effects of NT were evaluated by measuring its influence on several crucial factors in inflammatory pathways, including total antioxidant activity, antinociceptive effects *in vivo*, nuclear factor kappa B (NF-κB), polymorphonuclear cells (PMN), inducible nitric oxide synthase (iNOS), adhesion molecule (ICAM-1) and tactile hyperalgesia.

**Results:**

NT treatment decreased the levels of tumor necrosis factor alpha (TNF-α) and interleukin 1 beta (IL-1β). NT treatment decreased the total antioxidant status (TAOS) and reduced CFA-induced tactile hyperalgesia in a dose-dependent manner. NT significantly inhibited regulation of NF-kappaB activation and the production of IL-1β, TNF-α, iNOS and CAM-1. Moreover, NT suppressed infiltration of PMN.

**Conclusions:**

Our results showed that NT reduced CFA-induced tactile hyperalgesia through inhibition inflammatory pathways in experimental inflammatory rats.

## Background

Snake venoms are composed mainly of proteins and peptides, which possess a variety of biological activities. Most of snake venoms have demonstrated antinociceptive activity, and certain isolated neurotoxins have demonstrated significant analgesia in animal models [1,2]. Neurotoxin-Nna (NT), an analgesic peptide separated from the venom of Naja naja atra [3,4], is endowed an exceptional specificity of action that block transmission of the nerve impulse by binding to the α- subunit of the nicotinic acetylcholine receptor in the membrane [5-7].

Pain is often associated with inflammation, which results from tissue damage, chemical stimuli or autoimmune processes. These stimuli induce the release of inflammatory mediators (prostaglandins, bradykinin, histamine, growth-factors and neurogenic factors) [8]. These processes can lead to central sensitization and hypersensitivity. The antinociceptive effects of neurotoxins from snake venoms of Naja naja atra have been reported [1,2]. NT, one of the main components in the venom of Naja naja atra, produced significantly analgesic effects in animal model. However, there is little information available about the anti-inflammatory effects of NT. We undertook the present study to ascertain whether NT has the anti-inflammatory effects in rats.

## Methods

### Animals

Healthy male Wistar rats (2 months old and weighing 225 ± 25 g) and female Kunming strain mice (weighing 20-22 g) were purchased from the Experimental Animal Center of Zhejiang Chinese Medical University. The study was approved by Zhejiang University’s ethics committee, and all animal experiments followed the Guidelines published by the Ministry of Science and Technology of the People's Republic of China. Care was taken to minimize discomfort, distress, and pain to the animals.

### Chemicals

NT, an analgesic peptide separated from the venom of Naja naja atra [3,4], was provided by Kunming Institute of Zoology, the Chinese Academy of Sciences.

### Experimental design

#### Anti-hyperalgesia effect of NT in mice

Ten mice received a single intraplantar injection of 100 μl of 1 mg/ml dose of heat-killed and dried Mycobacterium tuberculosis in a mixture of paraffin oil and mannide monoleate. The tactile hyperalgesia was tested as tactile withdrawal threshold before and 15, 30 and 60 min after drug administration.

#### Anti -inflammatory effects in rats

Thirty-six rats were randomly divided into six groups: (1) animals were treated only with intrapleural injection (i.p.) of sterile saline (NaCl 0.95%) (Control group), (2) animals were treated with carrageenan (i.p.) and orally administrated with saline 10 ml/kg (Inflammatory group), (3) animals were treated with carrageenan (i.p.) and orally administrated with diclofanac 1.7 mg/kg (Diclofanac group), (4) animals were treated with carrageenan (i.p.) and orally administrated with NT 1.0 mg/kg (NT-1 group), (5) animals were treated with carrageenan (i.p.) and orally administrated with NT 2.0 mg/kg (NT −2 group) and (6) animals were treated with carrageenan (i.p.) and orally administrated with NT 4.0 mg/kg (NT-4 group). The NT (1.0, 2.0 or 4.0 mg/ kg body wt.), diclofanac sodium (1.7 mg/kg) and vehicle (saline 10 ml/kg) were administrated with respective drugs 1 h before the injection of carrageenan. Inflammatory was induced by a single intrapleural injection of 0.1 mL of sterile saline (NaCl, 0.95%) plus carrageenan (Cg, 1%). Six hours after the injection of carrageenan, blood samples were collected from orbital vein in all rats and serum was separated for biochemical estimations.

#### Measurement of IL-1β, TNF-α level and activation of NF-κB

The serum concentration of tumor necrosis factor alpha (TNF-α) and interleukin 1 beta (IL-1β) were measured using a commercial enzyme-linked immunosorbent assay (ELISA) kit (Shanghai Jinma Biological Technology, Inc., China) following the manufacture’s instruction. Activated Nuclear factor kappa B (NF-κB) was tested via ELISA-technique using the PathScan Phospho-NFκB p65 (Ser536) Sandwich ELISA Antibody Pair (Shanghai Yubo Biological Technology, Inc., China), following the manufacture’s instruction. Briefly, the activated NF-κB specifically binds to oligonucleotides corresponding to NF-κB consensus binding sites and immobilized in a 96-well plate. Bound NF-κB is detected with an anti-p65 antibody. Addition of a secondary HRP-conjugated antibody provides sensitive colorimetric readout quantified by spectrophotometry at 450 nm.

#### Measurement of Maleic dialdehyde (MDA)

Maleic dialdehyde (MDA), a reliable marker for lipid peroxidation, was measured using a thiobarbituric acid assay (Nanjing Jiancheng Bioengineering Institute) according to the manufacturer’s instructions. The total protein content of the samples was measured using a Coomassie Blue assay (Nanjing Jiancheng Bioengineering Institute) and the MDA content (nmol/mg protein) calculated using the following formula: absorbance of sample tube/absorbance of standard tube × 2.5 [9,10].

#### Measurement of infiltration of PMN

Meloperoxidase (MPO) activity was measured to assess the extent of PMN infiltration. The samples were stored at −70°C until assay for MPO. The method of assaying MPO activity was according to the guide of the assay kit (Nanjing Jiancheng Bioengineering Co Ltd, China).

#### Measurement of iNOS and ICAM-1 level

The procedures were processed according to the protocols recommended for inducible nitric oxide synthase (iNOS) and adhesion molecule (ICAM-1) immunohistochemistry kit (Hengdabaisheng Biotechnology, Beijing, China). The samples were exposed to 3% hydrogen peroxide for 10 minutes to bleach endogenous peroxidases. Then microwave oven-based antigen retrieval was performed. Slides were probed with either anti-ICAM-1 (1 :100, rat monoclonal, Hengdabaisheng Biotechnology, Beijing, China) and anti-iNOS (1:100, rat polyclonal, Hengdabaisheng Biotechnology, Beijing, China for 1 hour at 37°C, washed 3 times in PBS, incubated with biotin-labeled anti-rat IgG for 1 hour at 37°C, respectively. Incubation with PBS instead of the primary antibody served as a negative control. After washing in PBS, samples were visualized with 3, 3^‘^ -diaminobenzidine tetrahydrochloride (DAB) and counterstained with hematoxylin. Finally, the samples were dehydrated in graded ethanol, immersed in xylene and coverslipped. In specimens the positive cells were counted in ten randomly selected areas from each case and expressed as number of immunopositive/mm^2^.

#### Measurement of total antioxidant status

The total antioxidant status (TAOS) of serum was determined as previously described by Laight et al. [11]. The increase of absorbance at 405 nm was measured by a microplate reader (Shanghai Xunda Medical Technology, Inc., China).

### Statistical analysis

The data are shown as the mean ± SEM. All data were analyzed by a one-way analysis of variance, and the differences between means were established by Duncan’s multiple-range test. The significant level of 5% (*P < 0.05*) was used as the minimum acceptable probability for the difference between the means.

## Result and discussion

### Anti-inflammatory effect of NT on cytokines levels

**Figure 1:**
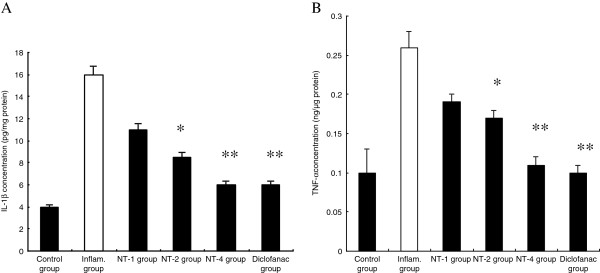
**Effect of NT on cytokines levels.** Inflammatory reaction induced by carrageenan had significantly elevated interleukin-1β (IL-1β) (**A**) and tumour necrosis factor-α (TNF-α) (**B**), and this elevation is significantly attenuated by the administration of NT (n = 6). Values are shown as means ± SEM. *p < 0.05 vs. inflammatory group, **p <0.01 vs. inflammatory group.

Cytokines are small glycoproteins produced in response to an antigen, and were originally described as mediators for regulating the innate and adaptive immune responses. IL-1β and TNF-α are of particular importance because they play a major role in coordinating mechanisms that command pro-inflammation [12,13]. The suppression of these cytokine has been found to reduce the severity of the inflammatory reaction. Figure 1 showed that inflammatory reaction induced by carrageenan significantly increased protein concentration of IL-1β and TNF-α (*P* < 0.01). NT treatment decreased the level of IL-1β and TNF-α in a dose-dependent manner.

### Effects of NT on NF-κB activation

**Figure 2:**
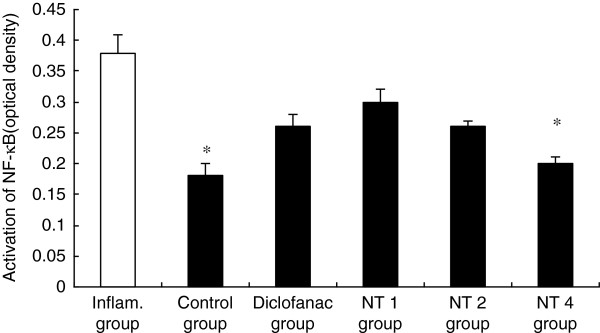
**Effect of NT on activation of NF-κB.** Inflammatory reaction induced by carrageenan had significantly elevated activation of NF-κB, and this elevation is significantly attenuated by the administration of NT-4 (n = 6). Values are shown as means ± SEM. *p < 0.05 vs. inflammatory group.

NF-κB comprises a family of transcription factors that act as regulators of pro-infammatory mediators [14]. NF-κB activation is correlated with significant increases in IL-1β and TNF-α mRNA levels [15]. Therefore, we hypothesized that NT may potentially show beneficial effects by decreasing the expression of NF-κB. As shown in Figure 2, carrageenan significantly induced activated NF-κB above control levels (*P* < 0.01), and as hypothesized, NT significantly suppressed this response in a dose-dependent manner.

### Effects of NT on Maleic dialdehyde (MDA)

**Figure 3:**
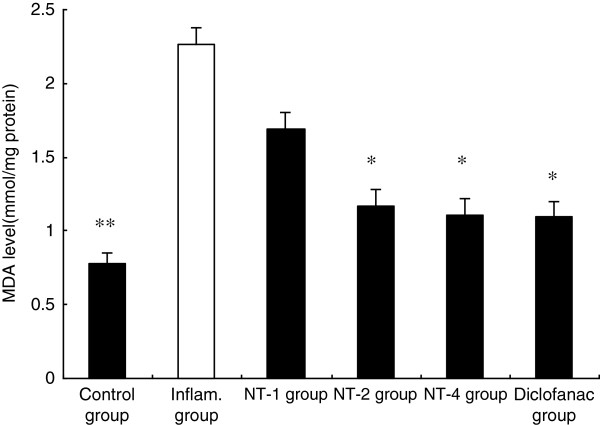
**Effect of NT on MDA level.** Inflammatory reaction induced by carrageenan had significantly elevated MDA level, and this elevation is significantly attenuated by the administration of NT (n = 6). Values are shown as means ± SEM, *p < 0.05 vs. inflammatory group. **p <0.01 vs. inflammatory group.

\Peroxidation damage plays an important role in the progression of LPS-induced injury. Therefore, the anti-oxidant effects of NT were investigated by measuring MDA levels. The control animals showed low MDA levels. However, the MDA levels in the saline group were significantly higher (*P* < 0.05). As shown in Figure 3, MDA level in the NT-2 and NT-4 groups were significantly lower than those in the saline group (both *P* < 0.05).

### Effects of NT on infiltration of PMN

**Figure 4:**
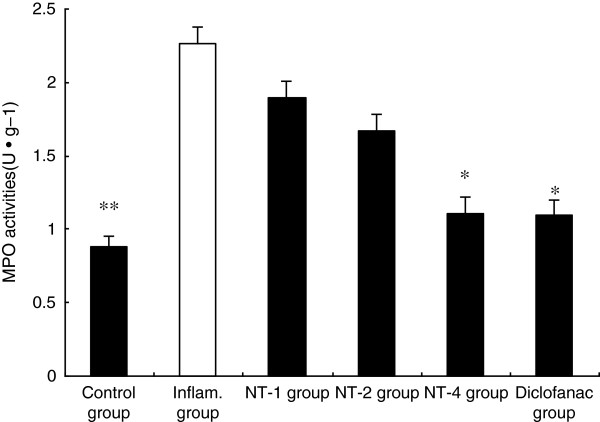
**Effect of NT on MPO activities.** Inflammatory reaction induced by carrageenan had significantly elevated MPO activities, and this elevation is significantly attenuated by the administration of NT-4 (n = 6). Values are shown as means ± SEM. *p < 0.05 vs. inflammatory group, **p <0.01 vs. inflammatory group.

PMN infiltration is initiated after generation of inflammatory mediators. The activity of MPO was determined as an indicator of PMNs migration. As shown in Figure 4, the MPO activity was relatively low in control group. Carrageenan treatment significantly increased the MPO activity compared with the control group (*P* < 0.01). Treatment with NT-4 significantly reduced MPO activity (*P* < 0.05). Treatment with NT-2 and NT-1 also reduced MPO activity. However, there was no difference among NT-1, NT-2 and saline group.

### Effects of NT on iNOS and ICAM-1 level

Rats subjected to inflammatory process induced by carrageenan showed typical markers of inflammation including upregulation of adhesion molecule and induction of prooxidative enzymes (Table 1). Inducible NOS is induced in response to inflammatory-like stimuli and is capable of sustained production of high levels of NO that predominate during inflammation [16]. The excessive or inappropriate production of NO can damage tissue through the superoxide anion (O_2_^−^) [17]. NT treatment dose-dependently decreased the protein expressions of iNOS (Table 1).

**Table 1:** **Effect of NT on ICAM-1and iNOS (number of immunopositive/mm**^**2**^**)**

**Different groups**	**iNOS**	**ICAM-1**
Control	10.41 ± 3.88 **	22.22 ± 6.22 **
Inflammation	68.28 ± 9.30	135.11 ± 21.35
NT-1	53.22 ± 1.22	96.41 ± 21.23
NT-2	44.22 ± 1.20*	80.41 ± 21.11*
NT-4	26.36 ± 2.33**	68.41 ± 33.32**
Diclofanac sodium	20.30 ± 1.59 **	59.36 ± 26.30 **

Values are shown as means ± SEM. *p < 0.05 vs. inflammatory group, **p <0.01 vs. inflammatory group.

Among the immunoglobulin family member, ICAM-1 has been the most extensively investigated in inflammatory process. Patients with acute inflammation had higher soluble ICAM-1 levels compared to patients without disease [10]. The protein expressions of ICAM-1 in the inflammatory group significantly increased compared with those of the control group. NT-2 and NT-4 treatment markedly decreased the level of ICAM-1 as compared to the saline group. (*P* < 0.05 and *P* < 0.01, respectively) (Table 1).

### Effects of NT on total antioxidant status

The total antioxidant status (TAOS) is an indication of O_2_^−^ and other oxidant species. We measured TAOS activity as an indirect indication of the formation of O_2_^−^ and other oxidant species. The NT-4 group had the lower level of TAOS activity in comparison to the inflammatory group (*P* <0.01) (Table 2). O_2_^−^ is produced by polymorphonuclear leukocytes and macrophages from the enzyme activity of NADPH oxidase and xanthine oxidase at inflammatory sites. We hypothesized that NT exert an anti-inflammatory effect through decreasing the levels of TAOS activities.

**Table 2:** Effect of NT on TAOS activity (μM L-ascorbate)

**Different groups**	**TAOS activity (μM L-ascorbate)**
Control	28.41 ± 3.10 **
Inflammation	79.33 ± 7.32
NT-1	63.22 ± 2.22
NT-2	48.02 ± 4.22*
NT-4	36.36 ± 3.33**
Diclofanac sodium	35.00 ± 6.60 **

Values are shown as means ± SEM. *p < 0.05 vs. inflammatory group, **p <0.01 vs. inflammatory group.

### Anti-hyperalgesia effect of NT in animal model

**Figure 5:**
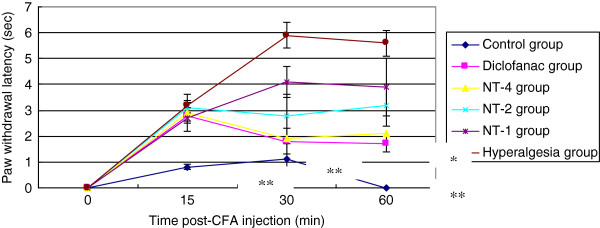
**Effect of NT on anti-hyperalgesia.** Hyperalgesia reaction induced by Mycobacterium tuberculosis was significantly attenuated by the administration of NT-4 (n = 10). Values are shown as means ± SEM. *p < 0.05 vs. hyperalgesia group, **p <0.01 vs. hyperalgesia group. NT has important effects of antihyperalgesic activity in animal models of inflammatory pain.

Complete Freund’s adjuvant (CFA) -induced hyperalgesia is frequently used as an animal model to study chronic inflammatory pain. The CFA-induced inflammation is accompanied by a tactile hyperalgesia (HA), which is robust over several days [18]. 4 mg/kg NT treatment significantly reduced the CFA-induced tactile hyperalgesia (*P* < 0.05) (Figure 5). It is likely that the antihyperalgesic effect of NT was due to a genuine anti-inflammatory effect.

## Conclusion

The results demonstrated here provide new evidence that NT has important effects of anti-inflammatory, antioxidant, peripheral antinociceptive and antihyperalgesic activity in animal models of inflammatory pain. The data suggest that NT is a potent anti-inflammatory and analgesic medicine.

## Competing interests

The authors declare that they have no competing interests.

## Authors’ contributions

YR carried out all of the studies in the methods section. LY performed the analysis of collected data. BZ drafted the manuscript. JG conceived of the study, participated in its design and coordination and helped in drafting of the final manuscript. All authors read and approved the final manuscript.

## Pre-publication history

The pre-publication history for this paper can be accessed here:

http://www.biomedcentral.com/1472-6882/13/86/prepub
